# Prevalence of burnout among healthcare workers in six public referral hospitals in northeastern Brazil during the COVID-19 pandemic: a cross-sectional study

**DOI:** 10.1590/1516-3180.2021.0287.R1.291021

**Published:** 2022-06-06

**Authors:** Ana Irene Carlos de Medeiros, Rafael Barreto de Mesquita, Felipe de Souza Macêdo, Antonio George de Calvacante Matos, Eanes Delgado Pereira

**Affiliations:** I PT. Doctoral Student, Universidade Federal do Ceará (UFC), Fortaleza (CE), Brazil.; II PT, PhD. Adjunct Professor, Department of Physiotherapy, Universidade Federal do Ceará (UFC), Fortaleza (CE), Brazil.; III MD, MSc. Physiotherapist, Department of Clinical Medicine, Universidade Federal do Ceará (UFC), Fortaleza (CE), Brazil.; IV MD, PhD. Assistant Professor, Department of Clinical Medicine, Universidade Federal do Ceará (UFC), Fortaleza (CE), Brazil.; V MD, PhD. Adjunct Professor, Department of Clinical Medicine, Universidade Federal do Ceará (UFC), Fortaleza (CE), Brazil.

**Keywords:** Burnout, professional, Intensive care units, Covid-19, Burnout syndrome, Prevalence, Healthcare workers

## Abstract

**BACKGROUND:**

The coronavirus disease 2019 (COVID-19) pandemic has placed considerable psychological stress on frontline healthcare workers (HCWs).

**OBJECTIVE:**

To evaluate the prevalence of burnout syndrome among HCWs facing the COVID-19 outbreak.

**DESIGN AND SETTING:**

Cross-sectional study conducted in six public intensive care units (ICUs) in the city of Fortaleza, Brazil.

**METHODS:**

An online survey was conducted among HCWs to measure the three dimensions of burnout.

**RESULTS:**

A total of 62 physicians (23.4%), 65 nurses (24.5%), 58 nurse technologists (21.9%) and 80 physiotherapists (30.2%) completed the questionnaire. Nearly half of the participants (48.6%) had high levels of emotional exhaustion, and almost one-third of them (29.4%) had high levels of depersonalization. Low levels of professional efficacy were observed in 18.1% of the sample. The independent determinants of depersonalization burnout were age < 33 years (odds ratio, OR 2.03; 95% confidence interval, CI 1.15-3.56; P = 0.01) and female gender (OR 0.33; 95% CI 0.18-0.62; P = 0.01). Increased workload was associated with both depersonalization (OR 2.37; 95% CI 2.02-5.50; P = 0.04) and emotional exhaustion (OR 1.89; 95% CI 1.04-3.58; P = 0.030).

**CONCLUSION:**

The COVID-19 pandemic has had a great impact on the dimensions of depersonalization and emotional exhaustion. Consideration of these dimensions is important when designing future burnout prevention programs for frontline personnel.

## INTRODUCTION

Burnout syndrome is defined as a set of psychological symptoms resulting from the interaction between chronic occupational stress and individual factors. These symptoms include emotional exhaustion, depersonalization and decreased professional satisfaction. Maslach and Jackson created the Maslach Burnout Inventory (MBI), which is currently the most commonly used scale for assessing the syndrome.^1^

The impact of the coronavirus disease 2019 (COVID-19) pandemic on frontline healthcare workers (HCWs) has been enormous and has resulted in high prevalence of burnout.^2–4^ This pandemic has exacerbated stressors at workplaces and increased occurrence of burnout syndrome among HCWs.^5^ A study on HCWs in Italy showed that at least one out of three exhibited high levels of the domain of emotional exhaustion, and one out of four reported high levels of the domain of depersonalization.^6^

The MBI is composed of three domains: emotional exhaustion, depersonalization and personal fulfillment. There is a lack of consensus regarding whether high scores are needed in one, two or all three domains to be able to state that HCWs are classified as burned out or non-burned out.^7,8^

It is recommended that each of the three MBI domains should be evaluated because the symptoms differ between individuals, and exhaustion can manifest itself as cynicism or anger in some and withdrawal and silence in others. Absence of any of the domains can lead to an erroneous assessment of the problem and consequent errors in healthcare policies and actions.

## OBJECTIVE

We conducted an online survey among HCWs at six public intensive care units (ICUs) in the city of Fortaleza, Brazil, in order to document the prevalence of each domain of burnout and the factors associated with these domains, during the COVID-19 outbreak.

## METHODS

In this cross-sectional study, we measured the prevalence of burnout among HCWs facing the COVID-19 pandemic during June and July 2020, while working in six public ICUs in tertiary-level referral hospitals for treatment of COVID-19 in the city of Fortaleza. We selected HCWs such that the sample included physicians, nurses, nurses technologists and physiotherapists. Anonymity of all the participants was guaranteed. All participants read and signed a written consent statement so that we could ensure that they understood the terms and agreed to participate in the study. The ethical procedures of the study were analyzed and approved by the independent Ethics Committee of the Universidade Federal do Ceará on February 27, 2019, under the number 04582818.6.00005054. This research project was written and approved for evaluation of burnout and its correlation with resilience among healthcare professionals working in ICUs in 2019. Thus, the project was designed before the beginning of the COVID-19 pandemic. However, with the onset of the pandemic, we decided to apply the burnout questionnaire online among healthcare workers in ICUs.

The survey was conducted through a questionnaire built on the Google platform and sent out through social networks. The questionnaire included questions about the participants’ demographic characteristics (age, gender and marital status), professional history (job category and years of experience), work characteristics (average weekly hours worked, how many hospitals they worked at, etc.) and habits (drinking alcohol, etc.).

The prevalence of burnout among HCWs in the ICU was measured using the Brazilian version of the Maslach Burnout Inventory-Human Service Survey (MBI-HSS).^9,10^

The questionnaire consisted of 22 questions: five items on depersonalization, nine items on emotional exhaustion and eight items on reduced professional satisfaction. The score for each item in the MBI-HSS was obtained using a seven-point Likert scale, which ranged from zero (never) to six (every day). The results were determined by summing the scores for each domain. This 22-item questionnaire contains three subscales that evaluate what are considered to be the three major domains of burnout. First, emotional exhaustion burnout is characterized by high scores (≥ 26). Second, depersonalization burnout is characterized by high scores (≥ 9). Third, professional efficacy burnout is characterized by low scores (≤ 33).^9^

### Sample size

Previous studies showed that the prevalence of burnout among HCWs ranged from 6% to 47%.^11^ To compare the rates of burnout between physicians and other HCWs, we assumed rates of 15% and 30%, respectively. By defining α and β as 0.05 and 0.20, respectively, at least 133 participants were required for one arm of the study, i.e. physicians versus other HCWs.

### Data analysis

Data from Google Forms were exported to a Microsoft Excel 2016 spreadsheet (Microsoft, Redmond, Washington, United States), and all statistical analyses were performed using SPSS Statistics, version 26.0 (SPSS Inc., Chicago, Illinois, United States). Absolute and relative frequencies (n, %) were used to describe the categorical variables. Continuous variables were described as medians and interquartile ranges.

First, we compared the baseline characteristics of those who did and did not have each domain of burnout using the χ^2^ difference test for categorical variables. Normality was verified using the Shapiro-Wilk test.

Logistic regression analyses were then performed using emotional exhaustion (≥ 26; yes/no), depersonalization (≥ 9; yes/no) and professional efficacy (≤ 33; yes/no) as dependent variables. The association between each burnout domain and each potential risk factor was explored by estimating the odds ratio (OR) and 95% confidence interval (CI), using bivariate analysis. The factors were selected *a priori* on clinical or empirical grounds, or were derived from the relevant literature. Predictors presenting α < 0.05 in the bivariate analysis were included in the multivariable logistic regression model.

## RESULTS

A total of 265 HCWs completed the questionnaire. The participants included 62 physicians (23.4%), 65 nurses (24.5%), 58 nurse technologists (21.9%) and 80 physiotherapists (30.2%). Nearly half of the participants (48.6%) had high levels of emotional exhaustion, and almost one-third of them (29.4%) had high levels of depersonalization. Low levels of professional efficacy were observed in 18.1% of the sample (Table 1).

**Table 1.** Characteristics of the sample

	n	%
**Characteristics**		
Women	204	67
Age in years, median (interquartile range)	33 (29-38)	
Age < 33 years	127	48
Married	143	54
**Professional history**		
Physicians	62	23.4
Other healthcare workers	203	76.6
**Occupation**		
Physicians	62	23.4
Nurses	65	24.5
Nurse technologists	58	21.9
Physiotherapists	80	30.2
Working in ≥ 2 hospitals	106	40
Increased workload	215	81.1
Increased income source	213	80.4
Increased drinking of alcohol	72	27.2
Working for more than 30 hours/week	246	92.8
Length of experience less than six years	200	75.5
**Burnout subdomains**		
Emotional exhaustion	129	48.6
Depersonalization	78	29.4
Professional efficacy	48	18.1

The emotional exhaustion group had more respondents with increased workload than did the group without emotional exhaustion (86% versus 76.4%; P = 0.04) (Table 2). The depersonalization group had a significantly higher number of physicians (32% versus 19%; P = 0.03), a lower number of women (62.8% versus 82.9%; P = 0.01), a higher number of professionals younger than 33 years old (61.5% versus 42.1%; P = 0.04), a higher number of unmarried professionals (57.7% versus 41.2%) and higher numbers of HCWs who were working in two or more hospitals (52.6% versus 34.5%; P = 0.007) and with increased workload (89.7% versus 77.5%; P = 0.02), compared with the group without depersonalization (Table 3).

**Table 2.** Comparison of characteristics of participants with and without emotional exhaustion

Characteristics	With emotional exhaustion (129) n (%)	Without emotional exhaustion (136) n (%)	Overall (265) n (%)	P
Women	102 (75)	102 (79)	204 (77)	0.43
Age < 33	66 (51.2)	61 (44.9)	127 (47.9)	0.40
Married	76 (58.9)	67 (49.3)	143 (54)	0.15
**Professional history**				
Physicians	32 (24.8)	30 (22.1)	62 (23.4)	
Other healthcare workers	97 (75.2)	106 (77.9)	203 (76.6)	0.57
**Occupation**				
Physician	32 (24.8)	30 (22.1)	62 (23.4)	
Nurse	29 (22.5)	36 (26.5)	65 (24.5)	
Nurse technologist	21 (16.3)	37 (27.2)	58 (21.9)	
Physiotherapist	47 (36.4)	33 (24.3)	80 (30.2)	0.05
Working in ≥ 2 hospitals	57 (36)	49 (44.2)	106 (40)	0.17
**Changes in relation to pre-pandemic period**				
Increased workload	111 (86)	104 (76.4)	215 (81.1)	0.04
Increased income source	99 (76.7)	114 (83.8)	213 (80.4)	0.14
Increased drinking of alcohol	32 (24.8)	40 (29.4)	72 (27.2)	0.40
Working than 30 hours/week	122 (91.2)	124 (94.6)	246 (92.8)	0.28
Length of experience less than six years	96 (74.4)	104 (76.5)	200 (75.5)	0.69

**Table 3.** Comparison of characteristics of participants with and without depersonalization

Characteristics	With depersonalization (78) n (%)	Without depersonalization (187) n (%)	Overall (265) n (%)	P
Women	49 (62.8)	155 (82.9)	204 (77)	0.01
Age years < 33	48 (61.5)	79 (42.2)	127 (49.7)	0.04
Married	33 (42.3)	110 (58.8)	122 (46)	0.01
**Professional history**				
Physicians	25 (32)	37 (19.0)	62 (23.4)	
Other healthcare workers	53 (67.9)	150 (80.2)	203 (76.6)	0.03
**Occupation**				
Physician	25 (32.1)	37 (19.8)	62 (23.4)	
Nurse	14 (17.9)	51 (27.3)	65 (24.5)	
Nurse technologist	17 (21.8)	41 (21.9)	58 (21.9)	
Physiotherapists	22 (28.2)	58 (31)	80 (30.2)	0.13
Work in ≥ 2 hospitals	41 (52.6)	65 (34.5)	106 (40)	0.007
**Changes in relation to pre-pandemic period**				
Increased workload	70 (89.7)	145 (77.5)	215 (81.1)	0.02
Increased income source	66 (84.6)	147 (78.6)	213 (80.4)	0.26
Increased drinking of alcohol	25 (32.5)	47 (25.1)	72 (27.2)	0.27
Working more than 30 hours/week	77 (98.7)	169 (90.4)	246 (92.8)	0.01
Length of experience less than six years	59 (75.6)	141 (75.4)	200 (75)	0.96

A multiple logistic regression analysis showed that depersonalization burnout among women was lower than that among men (OR 0.33; 95% CI 0.18-0.62; P = 0.01) and was higher among professionals younger than 33 years old (OR 2.03; 95% CI 1.15-3.56; P = 0.01) P = 0.01) **(** Table 4 **)**. Increased workload was associated with both depersonalization (OR 2.37; 95% CI 2.02-5.50; P = 0.04) and emotional exhaustion (OR 1.89; 95% CI 1.04-3.58; P = 0.030). No factors were associated with professional efficacy (Table 5).

**Table 4.** Logistic regression on factors associated with burnout subscales

Variables	Emotional exhaustion OR (95% CI)	Depersonalization OR (95% CI)
**Gender**		
Male		Reference
Female		0.33 (0.18-0.62)
**Age in years**		
≥ 33		Reference
< 33		2.03 (1.15-3.56)
**Increased workload**	1.89 (1.04-3.58)	2.37 (2.02-5.50)

OR = odds ratio; CI = confidence interval.

**Table 5.** Comparison of characteristics of participants with and without professional efficacy

Characteristics	With professional efficacy (217) n (%)	Without professional efficacy (48) n (%)	Overall (265) n (%)	P
Women	165 (76)	39 (81.3)	204 (77)	0.43
Age < 33 years	104 (47.9)	23(47.2)	127 (49.3)	0.99
Married	123 (56.7)	20 (41.7)	143 (54)	0.07
**Professional history**				
Physicians	49 (22.6)	13 (27.1)	62 (23.4)	
Other healthcare workers	168 (77.4)	35 (72.9)	203 (76.6)	0.50
**Occupation**				
Physician	49 (22)	13 (27.1)	62 (23.4)	
Nurse	53 (24.4)	12 (25)	65 (24.5)	
Nurse technologist	48 (22.1)	10 (20)	58 (21.9)	
Physiotherapists	67 (30.9)	13 (27)	80 (30.2)	0.90
Working in ≥ 2 hospitals	57 (44.2)	49 (36)	106 (40)	0.94
**Changes in relation to pre-pandemic period**				
Increased workload	173 (79.7)	42 (87.5)	215 (81.2)	0.21
Increased income source	173 (79)	40 (83)	213 (80)	0.56
Increased drinking of alcohol	64 (29.5)	8 (16.7)	72 (27.2)	0.07
Work more than 30 hours/week	199 (91.7)	47 (97.9)	246 (92.8)	0.13
Length of experience less than six years	161 (72.4)	39 (81.3)	200 (75)	0.30

## DISCUSSION

The results from this study were concordant with those from studies carried out in other countries.^3,4,12,13,14^ The proportions with emotional exhaustion, affecting nearly half (48.6%) of the HCWs, with depersonalization in almost one-third (29%) and with low levels of professional effectiveness in less than one-fifth (18%) were similar to the results found by Barello et al.^6^

The contributions of sociodemographic variables to the three burnout domains were explored using multivariate logistic regression analysis. Our findings suggested that female gender is associated with lower levels of depersonalization burnout. This can be explained by the burnout/resilience balance. Duarte et al.^3^ observed that resilience is a potentially protective factor against burnout. There is evidence that women are more resilient and have better coping skills, which in turn reduces work stress and allows them to deal with work-related issues more effectively.^15^

A recent meta-analysis on the relationship between gender and burnout showed that women are slightly more emotionally exhausted than men, while men are slightly more depersonalized than women.^16^

Lower age was associated with depersonalization burnout, which is in line with previous research.^3,4^ This result can at least partly be explained by the imbalance between expectations about attributions and the reality of the challenges and stressors of the ICU for young professionals. These HCWs, including junior doctors and residents, have formed an important pillar of the effort involved in managing and treating patients during the COVID-19 pandemic.^17^ Because of the large number of patients and the heavy burden of the COVID-19 pandemic, many healthcare units, including ICUs, have had to hire young HCWs who, contrary to what was previously thought, are not immune to burnout.

Increased workload is associated with depersonalization and emotional exhaustion. Our findings are consistent with previous research.^4^ Work overload is one of the most important risk factors for burnout among healthcare professionals.^18,19^ According to Leiter’s burnout model, there is evidence to suggest that emotional exhaustion caused by work overload may lead to depersonalization and cynical attitudes.^20,21^

There were no covariables associated with the HCWs’ performance. This can be explained by the fact that only 18.1% of the HCWs in the sample had low levels of performance. Empirical evidence points towards exhaustion and cynicism as the core of burnout.^22^ In our sample, these two domains had high prevalences.

This study had several limitations. The sample size actually achieved may have been insufficient. It was a cross-sectional online survey, which may have limited its accessibility for individuals with little or no skill regarding the internet. Because this was a cross-sectional study, no data were collected before the pandemic, thus making comparison impossible. Other variables, such as depression and anxiety, have not yet been studied. Longitudinal studies will be needed to clarify the long-term effects of physical and psychological variables on HCWs during the COVID-19 pandemic.

This is an important study that highlights the multidimensionality of burnout syndrome among several types of healthcare professionals in six public ICUs. Indeed, the two burnout domains were associated with specific variables. Previous studies have emphasized the importance of identifying potential factors leading to burnout among HCWs in order to be able to implement remedies for management and prevention of burnout syndrome. These interventions should be at both the individual and the organizational level, and could involve scheduling of activities to enable a healthy work-life balance, strengthening of relationships with family and friends, fulfillment of personal goals and ensuring organizational support from the hospital.^23^

We believe that the results from this study contribute to better understanding of the factors associated with burnout among HCWs and should be considered in designing future programs and guidelines to promote protective actions and increase the psychological wellbeing of these professionals. The idea of “burnout contagion” can be useful for “emotional decontamination” in workplaces, among workers who have already been affected by this syndrome.

## CONCLUSION

HCWs experience high levels of emotional exhaustion and depersonalization burnout, which warrant attention and support from policymakers.
